# Does space matter? Estimation and evaluation of required space for commercial mass culture of grasshoppers (Acridoidea: Orthoptera)

**DOI:** 10.1371/journal.pone.0265664

**Published:** 2022-06-03

**Authors:** Amlan Das, Dipsikha Ghosh, Balaram Manna, Avishek Dolai, Anshuman Pati, Sumit Mandal, Krishnendu Mukherjee

**Affiliations:** 1 Department of Zoology, Entomology Laboratory, University of Calcutta, Kolkata, West Bengal, India; 2 Wildlife Institute of India, Dehradun, Uttarakhand, India; University of California Riverside, UNITED STATES

## Abstract

A space-dependent mortality assay was performed on thirty-one short-horned adult grasshopper species (Acridoidea: Orthoptera) to estimate the space required for mass culture of acridids in captivity. Our findings show that acridids have a multidimensional mortality mode at different densities. The correlations between density and mortality of acridids in rearing units follow a sigmoidal curve. Acridid mortality significantly increases with individual numbers up to a threshold, after which mortality does not change even if the density increases further. A log-logistic sigmoidal function expresses the dose (density)-response (mortality) relationship in the majority of acridid species. Mortality of acridids at variable densities does not necessarily correspond with the body-mass of the insects, indicating that mortality is a body-mass independent event. As a ready reference, a utility chart has been prepared, providing the necessary conversion factor for estimating space for a given number of acridids. The present information will be helpful for commercial grasshopper farming in captivity.

## Introduction

Several attempts have been made in recent years to develop sustainable methods for mass-rearing, a variety of insects for food and feed formulation for animal husbandry [1]. Three insect groups, namely crickets (Orthoptera: Gryllidae), mealworms (Coleoptera: Tenebrionidae), and black soldier fly larvae (Diptera: Stratiomyidae) are primarily accepted and cultured in some parts of the world [2]. Besides these, short-horned grasshoppers and locusts (Orthoptera: Acrididae) have recently gained significant attention from farmers and insect-farm entrepreneurs [3–6] for their convincing quality of nutritional contents and energy reserves [7–9]. Among acridids, very few are recorded as agricultural pests but most of them significantly impact the environment, playing a balancing role in the food-web network among the trophic levels [10–12].

Acridids have received much attention in the insect biomass production industry because of their numerous advantages [13–15]. For example, they can produce a significant amount of biomass in short period than other insects due to their high reproductive potential [16, 17] and rapid breeding cycle [12, 18]. A majority of acridids are multivoltine [19–21], indicating their ability to produce biomass throughout the year [3]. Besides these advantages, the gregarious habit of acridids [21–23] allows them to survive in denser conditions in a given space than other insects [24–26]. It is reported that after only a few hours of crowding, a solitarious acridid will shift from avoiding others to demonstrating gregarious behavior. When the insects become attracted to others rather than repulsed by them, a positive feedback loop is generated that can lead an initially solitary population to the gregariousness phase, and therefore a less-dense population turns into more-dense within some hours. All of these factors allow for mass propagation of this mini-livestock in a small space and in a short period, expanding the scope of commercial grasshopper farming [19, 27, 28]. An integrated approach of acridid mass-culture and transforming its biomass into food and feed products would thus be a new realistic method for economic earnings for sustainable societal benefits [29–31].

Grasshopper biomass can be used as a supplementary animal-protein rich feed ingredient [32–34] or can be served as a restaurant delicacy eliminating all social prejudices [6, 35, 36]. Despite the enormous farming potentiality of acridids [16], information on commercial mass-rearing techniques and measures for these mini-livestocks are lacking [3]. However, numerous reports on its rearing have been added to the literature solely to test experimental hypotheses. Reports on bio-eco-chemo-physiology and life-cycle traits [37, 38] for several species of acridids demonstrate their breeding capabilities [16, 39], food preferences [40], nutritional profiles [6, 41], optimal rearing [42] and egg incubation [43, 44] conditions, and hatching performances [45], but information regarding space requirement for mass culture of grasshoppers are remarkably missing.

Conditions for rearing insects, particularly the density of individuals, may substantially impact production efficiency as it is evident that increasing density may significantly increase mortality [46]. Therefore, to maximize the production rate of insects, rearing efforts should optimize rearing density and insect growth while minimizing mortality [47]. As density-mortality correlation shapes population structure, the mortality of cultivable insects depends on the density of the cultivable population [48]. Hence, that particular maximum density is desirable for mass culture for insects in a given volume while keeping the mortality of cultivable individuals low [49]. Although the gregarious habit of acridids favors the survival of these insects in dense conditions, it is unclear what scale of density would ideally result in a lower mortality rate [50]. Therefore, the purpose of this study is to determine the effective space requirement for mass-culture of acridids under captivity. We attempt to optimize this study by conducting mortality tests on the selected grasshopper species based on the hypothesis that acridid mortality is positively correlated with increasing individual density. Therefore, the present study primarily addresses, a) the optimal space requirement for acridid culture.

Body mass is a frequently used quality in ecological and evolutionary research [51] and is a critical variable in community structure [52]. Body size [53] or metrics of body conditions [54] are indicators of the energetic states of the insects and are often used to identify factors that influence their survival capacity [55]. It is widely assumed that the ’higher body condition’ of the insect corresponds to the higher fat content of the individual [54]. And thereafter, individual body mass may, to a large extent, influence the longevity of the beholder [56]. Thus insects with a higher ’body condition index’ survived better than those in poorer conditions. In this context, an individual’s body size determines its survival capacity [55]. Considering these theories, the present investigation attempts to address the second inquiry, b) does the mortality rate of acridids correlate with their body mass index (BMI)?

We set these two goals (a, b) as both are interlinked and dependent to each other during mass culture of insects. However, the primary goal of the present study is restricted to estimate the rearing space under captivity. As a ready reference for the mass-culture of grasshoppers, a utility chart has been prepared to depict independent contrasts of required space for thirty-one studied species. Our projected chart provides necessary conversion factor for estimating space for a given number of grasshoppers. In other way, the projected chart will also assist in estimating the individual number to be cultured for a given space. Though our chart value addresses species-specific information, it provides an inclusive picture for other species in general. As space requirement for mass culture of insects is directly related to yield, the present investigation will help smooth propagation of insect mass culture, especially for short horned grasshoppers (acridids).

## Materials and methods

### Focal species

Thirty-one species of Indian short-horned grasshoppers (Acridoidea: Orthoptera) from 12 different subfamilies were chosen for this study (Table 1). The species were selected for their ubiquitous presence in nature, availability of an adequate number for our study, and easy handling for mass culture and laboratory rearing. Even though there are diverse species of grasshoppers in the field beyond our selected ones, we have chosen only those abundant in the field. This is because the selected species either have higher egg-laying abilities or a lower mortality than others, so they emerge more frequently in the wild. Higher oviposition frequency and low mortality rate—both are beneficial for a livestock culture. Another line of reasoning is that since most of our selected species are considerably bigger, they can produce better biomass (crop yield) when cultivated than smaller ones. Furthermore, the majority of the species are multivoltine, meaning they produce offspring multiple times per year. The species can be tamed in cages with minimum effort and have comparable eating preferences. So, the same or similar kind of food plants can be used for different species during culture. All these are advantageous for the mass-biomass-production of grasshoppers. We have restricted our experiment to only one family, Acrididae, for comparative analysis of space-dependent mortality assay.

**Table 1:** Adult acridid mortality (species, n = 31; family, n = 1; subfamily, n = 12) at different density levels (DL1 to DL5).

Acridid species	Family	Subfamily	BMI	Adult mortality %				
				DL1	DL2	DL3	DL4	DL5
*Acrida gigantea* (Herbst, 1786)	Acrididae	Acridinae	0.09	60±9	97.5±10	95±11	86.3±11	81±10
*Oxya japonica* (Thunberg, 1815)	Acrididae	Oxyinae	0.17	15±2	60±9	76.7±10	80±10	79±10
*Spathosternum prasiniferum prasiniferum* (Walker, 1871)	Acrididae	Odipodinae	0.22	0±0	40±7	61.7±10	65±9	65±9
*Oxya nitidula* (Walker, 1870)	Acrididae	Oxyinae	0.23	15±3	32.5±6	70±10	66.3±9	65±9
*Acorypha glaucopsis* (Walker, 1870)	Acrididae	Calliptaminae	0.24	15±2	65±9	81.7±11	83.8±11	77±10
*Leva indica* (Bolívar, 1902)	Acrididae	Gomphocerinae	0.24	15±3	37.5±8	73.3±9	67.5±7	69±9
*Hieroglyphus nigrorepletus* (Bolívar, 1912)	Acrididae	Hemiacridinae	0.24	15±2	57.5±9	80±9	81.3±9	76±8
*Phlaeoba infumata* (Brunner von Wattenwyl, 1893)	Acrididae	Acridinae	0.25	0±0	50±7	65±5	68.8±8	66±8
*Acrida exaltata* (Walker, 1859)	Acrididae	Acridinae	0.27	15±4	65±9	68.3±8	77.5±10	75±9
*Cyrtacanthacris tatarica* (Linnaeus, 1758)	Acrididae	Cyrtacanthacridinae	0.27	15±3	55±6	75±7	77.5±9	73±9
*Heteracris littoralis* (Rambur, 1838)	Acrididae	Eyprepocnemidinae	0.28	20±4	60±9	65±6	66.3±8	67±8
*Phlaeoba panteli* (Bolívar, 1902)	Acrididae	Acridinae	0.30	5±1	47.5±6	70±7	71.3±8	67±9
*Schistocerca gregaria* (Forskal, 1775)	Acrididae	Cyrtacanthacridinae	0.30	10±1	52.5±7	66.7±6	80±8	76±9
*Acrida turrita* (Linnaeus, 1758)	Acrididae	Acridinae	0.31	55±8	92.5±12	88.3±10	81.3±8	77±10
*Acrotylus humbertianus* (Saussure, 1884)	Acrididae	Oedipodinae	0.31	15±3	52.5±8	71.7±9	81.3±9	76±9
*Gesonula punctifrons* (Stal, 1861)	Acrididae	Oxyinae	0.32	15±4	60±10	75±9	85±8	80±10
*Gastrimargus africanus* (Saussure, 1888)	Acrididae	Oedipodinae	0.32	10±3	57.5±10	71.7±8	78.8±7	75±9
*Oxya hyla hyla* (Serville, 1831)	Acrididae	Oxyinae	0.32	5±2	55±7	73.3±8	78.8±8	76±9
*Oxya fuscovittata* (Marschall, 1836)	Acrididae	Oxyinae	0.33	10±3	52.5±6	76.7±9	82.5±9	81±10
*Oedaleus abruptus* (Thunberg, 1815)	Acrididae	Oedipodinae	0.37	30±7	60±8	53.3±6	72.5±7	64±9
*Trilophidia annulata* (Thunberg, 1815)	Acrididae	Oedipodinae	0.37	55±9	72.5±9	75±9	75±8	67±7
*Oxya velox* (Fabricius, 1787)	Acrididae	Oxyinae	0.38	10±2	62.5±6	80±10	82.5±10	82±9
*Catantops erubescens* (Walker, 1870)	Acrididae	Catantopinae	0.41	10±3	42.5±4	68.3±7	82.5±10	79±9
*Eyprepocnemis alacris alacris* (Serville, 1838)	Acrididae	Eyprepocnemidinae	0.41	10±2	47.5±4	65±7	76.3±9	75±6
*Eucoptacra binghami* (Uvarov, 1921)	Acrididae	Coptacrinae	0.43	5±1	47.5±5	56.7±5	80±11	76±6
*Hieroglyphus oryzivorus* (Carl, 1916)	Acrididae	Hemiacridinae	0.47	10±3	52.5±5	78.3±10	80±11	74±7
*Acrotylus insubricus* (Scopoli, 1786)	Acrididae	Oedipodinae	0.53	40±9	72.5±7	78.3±10	77.5±10	72±7
*Aiolopus thalassinus tamulus* (Fabricius, 1798)	Acrididae	Oedipodinae	0.55	10±2	45±4	66.7±9	83.8±9	76±8
*Aiolopus simulatrix* (Walker, 1870)	Acrididae	Oedipodinae	0.63	10±2	42.5±4	65±9	85±10	74±7
*Morphacris fasciata* (Thunberg, 1815)	Acrididae	Oedipodinae	0.67	45±8	70±7	70±9	73.8±9	68±6
*Stenocatantops splendens* (Thunberg, 1815)	Acrididae	Catantopinae	0.70	30±5	52.5±5	56.7±8	62.5±8	60±5

The species are arranged in ascending order of body mass index (BMI). BMI of a species is calculated by considering the average BMI of a male and a female. Mortality was calculated using data from three replicates and is represented as mean±SD.

### Collection of species

All experimental species (n = 31) were collected from the wild across several states from India, poured into aerated plastic bags and transported to the laboratory. In laboratory, they were caged in cabinets, and allowed to propagate following the rearing methods described previously [3, 19]. Only the advanced acridid nymphs (IIIrd instar onwards) were collected by net-sweeping (30 cm diameter) and handpicking from different ecological fields (agriculture, grassland, and deciduous forest) during different seasons. Species were collected when they became available in the wild and when such species were found in nature, we then tested that species. Therefore, our studies took place throughout three seasons, from 2017 to 2019. For example, the experiment with *Oxya fuscovittata* was carried out in June (summer), while the experiment with *Hieroglyphus oryzivorus* was conducted during October (winter). This was done because the respective species were abundant in the field during the summer and winter.

### Permission for collection of the specimen

Collecting insects and other invertebrates is permissible in all non-protected and many protected locations throughout India. It is also permissible to preserve, store, and keep insect and other invertebrate specimens. As we are dealing with grasshopper specimens from diverse non-protected areas such as agriculture fields, we do not require authorization for insect collecting and study.

### Species rearing

After preliminary observation, the assorted nymphs were separated into different insect rearing cabinets and acclimatized in semi-laboratory environmental conditions (28±2°C temperature, 70–80% relative humidity, 12L/12D photoperiod) up to adulthood. During assorting the nymphs, acridids may not have been correctly identified species-wise; therefore adult individuals were separated taxonomically when they attained adulthood. Wooden framed nylon gauged insect rearing cabinets (90 cm × 60 cm × 60 cm) were prepared to house the nymphs for rearing purposes. Nearly equal amounts (wet-weight) of fresh tender grasses (*Cynodon dactylon*, Poaceae and *Cyperus kyllingia*, Cyperaceae), collected from wild, were used in water-filled conical flasks (10–20 mL) for their food and were replaced as required. Moist sterilized sand-filled (boiled at 120±10°C, 30 min, tap-water) plastic containers (10 cm height × 6 cm diameter) and water-soaked cotton balls were placed inside the cabinets to keep the environment humid. Floors of the cabinets were cleaned regularly by odourless disinfectant (Sodium hypochlorite liquid solution, 5% in tap-water) to avoid any fungal growth and/or predatory attack. Nymphs were reared in cabinets up to adulthood. Adult acridids were taxonomically identified and considered for density-level mortality assays.

### Experimental setup

#### Preparation of density level conditions

Five specific rearing conditions were prepared in order to estimate the space requirement for rearing the acridids, each with a certain number of individuals kept in a variable volume of space. Such rearing conditions are referred to here as ’Density Level’ in this context (DL). As a result, for each of the 31 species, five DLs were created: DL1 (10 adults in 20L container), DL2 (10 adults in 10L container), DL3 (10 Adults in 5L container), DL4 (10 Adults in 2.5L container), and DL5 (10 Adults in 1.25L container). Each DL treatment was replicated three times. Up to three days old adult individuals were used for all experimental sets; over-aged adults beyond three days were avoided to elude issues of natural death of the insects. Thus, DLs were prepared by compromising only the space of the accommodating chamber (volume) without manipulating the individual number. The volume of the experimental container was compacted to 50% than the preceding one for each case to observe how the insect behaved in an orderly compressed space. Thus, the present experiment looks into how the volume of the cultural space affects the mortality of the insects. All experiments were carried out with new (non-experienced) adult individuals only, with the male to female ratio remaining constant throughout. Therefore, out of 10 adult participants for an experiment, 5 were males, and 5 were females. We purposely maintained an equal number of male and female participants because both genders would be present rather than one in the practice of mass-culture of grasshoppers. We housed an equal number of individuals from both sexes in all experimental settings since individuals are anticipated to have a nearly equal sex ratio upon hatching [57].

While designing this experiment, we also purposefully did not alter the number of the insects by keeping the same volume of the container. Instead, we chose the other option, where we maintained a fixed number of insects (n = 10) in the container and changed the volume of the container as a variable parameter. We did so in order to assess the ’effective space’ for acridid culture. Accommodating insects in large numbers in variable units can cause issues erroneous mortality accounts for several reasons. The combined body volume of insects, for example, reduces the effective volume of the culture container, which affects mortality. Again, taking a large number of insects may increase their tendency of cannibalism, which may also affect mortality. To avoid these concerns, we kept the individual number constant for each adjusted rearing container throughout the study.

#### Experimental conditions

All experiments were carried out in a laboratory setting (28±2°C temperature, 70–80% relative humidity, and 12L/12D photoperiod) but on different days from 2017 to 2019, subject to availability of the samples (fledgling adults) emerged from our stock culture. During the experiment, we only provided tender leaves of *C*. *dactylon* and *C*. *kyllingia* in equal amounts (wet-weight) in water-filled conical flasks (10–20 ml) for food, which were replaced every three days. Though grasshoppers make different choices to food plants when different resources are available, they have a general preference for grasses from the Poaceae and Cyperaceae families. Hence, we provided the same dietary sources (*C*. *dactylon* and *C*. *kyllingia*) as food to all species to avoid any issues with nutrition-linked survivability/mortality. The floor of each container was covered with wet-tissue paper, and the mouths were tightened with fine mesh. To keep the assay chamber moist, tap water was sprinkled thrice a day on the tissue paper. Each DL assay was run for 21 days for each species. We set the test period of 21 days to avoid the issue of their normal death and incorrect mortality calculations thereof. It is well documented that each of the present focal species has an average adult lifespan of more than 21 days [21]. Throughout the 21-day experiment, we maintained a nearly identical experimental setup, same food plants, and sanitary precautions for each DL replicate of all species.

### Data acquisition and analysis

#### Mortality calculation

Dead adults (if any) at each DL were observed daily throughout the experiment (21 days), and if noticed, they were removed from the containers and counted. After 21 days, all dead individuals (if any) for a specific DL were recounted, and the mortality percent was calculated using the formula:

$$Mortality\;percentage\;\left(M\%\right)\;=\frac{number\;of\;dead\;individuals\;recorded\;after\;the\;experiment\;period}{\text{total}\;\text{number}\;\text{of}\;\text{individuals}\;\text{taken}\;\text{for}\;\text{experiment}}\times 100$$

The M% was calculated considering pooled mortality data from three replicates of a specific DL (three replicas of DL1; three replicas of DL2, and so on) and the pooled number of total individuals considered for that DL assay. In our experiment, pooled number of total individuals from each DL was constant (n = 30) (for example, DL1: 10x3 = 30; DL2: 10x3 = 30, and so on). M% at each DL for each studied species was calculated separately.

#### Dose-response model

We analyzed our data using the ’dose-response model’ where dose-response findings are expressed in terms of mortality percent of the acridid species under exposure to different DL conditions. The ’dose-response model’ is the regression model, where the independent variable is referred to as ’dose’ (DL), and the dependent variable is referred to as ’response’ (adult mortality). Here, we, therefore, define the ’dose’ as ’requirement of space’ as DL represents as a function of space. As in the present study, five DL categories (DL1, DL2, DL3, DL4, and DL5) were prepared for each species, ’concentration value’ for a given ’space’ (viz., 20 L, 10 L, 5 L, 2.5 L, and 1.25 L) were calculated independently against the respective ’space’ (container volume) for each DL. The calculated ‘concentration value’ for respective five ’space’ are as follows: 0.5 (10/20 for DL1); 1.0 (10/10 for DL2); 2.0 (10/5 for DL3); 4.0 (10/2.5 for DL4) and 8.0 (10/1.25 for DL5). We define ’response’ (*y*) to a given dose (DL) as the ’mortality percentage’ for that dose. It indicates the extent to which mortality percentages (i.e., responses) are shown for a given dose. The log value of each concentration is plotted on the X-axis [DL1: log (0.5) = -0.30103; DL2: log (1.0) = 0.0; DL3: log (2.0) = 0.30103; DL4: log (4.0) = 0.60206; and DL5: log (8.0) = -0.90309, respectively].

To evaluate the generalised response, we scaled dose-response expressions using log-logistic sigmoid functions. The sigmoidal functions are used to interpret a general dose-response model curve as:

y=p*ln(10)×(A2−A1)

Here, *A*1 (bottom asymptote of the curve) = 1,

*A*2 (top asymptote of the curve) = 2, where (*A*1<*A*2)

Centre = [LOGx0, (*A*1+*A*2)/2] or LOGx0 = 1 (slope, *p* = 0.2). The log-logistic model parameters are as follows:

$$y=A1+\frac{A2-A1}{1+10^{\left(LOGx0-x\right)p}}$$

Where, *y* = observed response; *A*1 = min (*y* data, bottom asymptote); *A*2 = max (*y* data, top asymptote) (*A*1<*A*2). We set *A*1 = 0, and *A*2 = 100 for the present data because mortality percentage will remain within that range (0–100) (no death = 0; and all died = 100). The centre of the *x-y* curve is LOGx0 = *x* at *y =* 50; where *p* = slope of the curve (*p*>0) denotes the probability of obtaining the results.

The following mathematical equations are used to calculate the effective concentration (EC) values (derived parameters):

$$\text{EC}10=10^{\left(\text{LOGx}0\;–\;\text{log}\;\left(9\right)/\text{p}\right)}$$

$$\text{EC}20=10^{\left(\text{LOGx}0\;+\;\text{log}\;\left(0.25\right)/\text{p}\right)}$$

$$\text{EC}50=10^{\left(\text{LOGx}0\right)}$$

$$\text{EC}80=10^{\left(\text{LOGx}0\;+\;\text{log}\;\left(4\right)/\text{p}\right)}$$

and

$$\text{EC}90=10^{\left(\text{LOGx}0\;+\;\text{log}\;\left(9\right)/\text{p}\right)}$$

Where,

EC10 = Concentration that causes 10% reduction in the number of individuals (= 10% mortality);

EC20 = Concentration that causes 20% reduction in the number of individuals (= 20% mortality);

EC50 = Concentration that causes 50% reduction in the number of individuals (= 50% mortality);

EC80 = Concentration that causes 80% reduction in the number of individuals (= 80% mortality);

EC90 = Concentration that causes 90% reduction in the number of individuals (= 90% mortality).

Using the dose-response model described above, we calculated EC values and correlated the relation between density (dose) and mortality (response) for the studied 31 species of grasshoppers. For example, if 10 individuals are accommodated inside a 100 L space, the density will be 0.5 (individual/space = density, D); based on D, EC value for each dose (DL) was calculated.

#### BMI-mortality correlation

Body mass index (BMI) for each species was calculated using the formula

$$Body\;Mass\;Index\left(BMI\right)=\frac{Weight\;of\;the\;individual\;\left(mg\right)}{Square\;of\;the\;individual\;length\;\left(mm\right)}$$

The weight and length of the insects were measured on a live insect basis, and the BMI value is calculated as a function of body weight and size (length) following previous researches [58, 59]. BMI for each species was calculated using at least 30 individuals with an equal ratio of males and females. Therefore, BMI in Table 1 (each value) represents the average BMI of 15 males and 15 females of adult individuals of a species. Male and female weights and lengths and BMI are likely to vary widely among grasshoppers, even though, in some cases, BMI may not differ much between males and females [60]. In the current context, the BMI of a species is estimated by taking the average BMI of males and females into account (Table 1). We did this on purpose, even though there were ways to determine the BMIs for each sex and link them to mortality. Since the sex ratio of an individual tends to be fairly equal according to Fisher’s principle [61], we assumed that when farmers culture grasshoppers on a large scale, both males and females would be present in roughly equal ratios in the rearing cabinets [62]. In contrast, by displaying BMI data separately for males and females and linking them with mortality, it would encounter difficulties in associating space with BMI for a species, as the dual BMI (male and female independently) does not accurately reflect reality in culture cabinets since both sexes present there in almost equal force simultaneously. Therefore, equal consideration was given to male and female individuals in the anticipation that nearly equal numbers of male and female individuals would be present in rearing cabinets (commercial mass rearing units). At each DL, BMIs for each species were independently correlated with mortality percentage (M %).

#### Data analysis

The maximum-minimum standardization method was used to standardize and reduce the dimensionality of the data. We used the Shapiro-Wilk test to determine the overall normality of our data (mortality at different DLs). Out of five DLs value, three DLs rejects the normality, we performed non-parametric statistical tests. Kruskal–Wallis test was performed to distinguish the mortality responses among the DLs and find the variations of mortality through Dunn’s test.

The dose-response relationship model was analyzed and graphically displayed using a 2D scatter plot. Principal component analysis (PCA) was used to identify the relationships among the DLs variables with species mortality.

Based on the space-dependent mortality, hierarchical cluster analysis was performed, resulting in a cladistic data representation of the species. Objects in the dendrogram are linked together based on their similarity. Pearson correlation analysis was performed for find out the relationship among BMI and mortality of the species. All statistical analyses were carried out using software such as SPSS (ver. 25), OriginLab (ver. 2021b), and R (ver. 4.0.1) (R Development Core Team 2011).

## Result

### Space-dependent mortality

**Fig 1:**
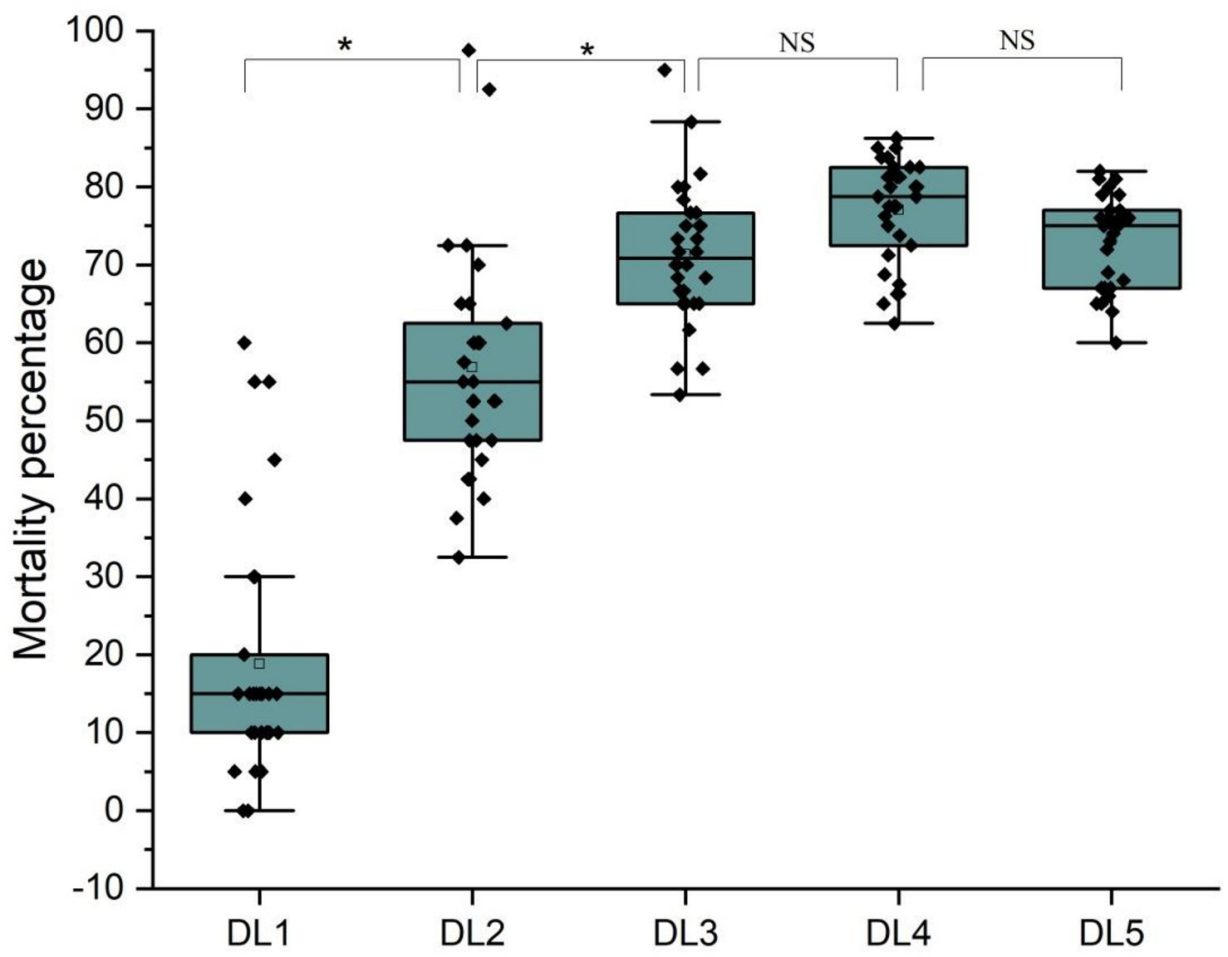
Mortality (M%) in Acridids at different density levels (DL1 to DL5). The upper and lower quartiles of each box represent the range of species mortality. M% for 31 species are represented by 31 dots in a box, where the line in the interquartile range represents the median value. Kruskal–Wallis test among DLs (*F*_4, 154_ = 146.133, *p* = 0.000, at 5%) reveals a significant difference in mortality across DLs. Dunn’s test analysis (0.05) reveals that M% differs significantly between DL1 and DL2 (*p* = 0.0) and DL2 and DL3 (*P* = 0.0), but not between DL3 and DL4 (*p* = 0.2894) and DL4 and DL5 (*p* = 0.63192).

Mortality percentages (M%) of acridids varied significantly (Kruskal–Wallis test, *F*_4, 154_ = 146.208, *p* = 0.000) with the change in DLs (from DL1 to DL5) amongst species (Table 1). The results show that acridid mortality increased significantly with decreasing space. Dunn’s test shows that M% increased significantly from DL1 to DL2 (*p* = 0.005) and from DL2 to DL3 (*p* = 0.005), but there were no significant differences in M% from DL3 to DL4 (*p* = 0.05) or from DL4 to DL5 (*p* = 0.05). This observation establishes a general idea that mortality of most acridids peaked at DL4, but after that, death rate of the species did not change significantly, even if the population number increased (Fig 1). Among the studied 31 species, mortality was 0–20% at DL1 (applicable for 24 species); 50–70% at DL2 (applicable for 16 species); 50–70% at DL3 (applicable for 14 species); >70% at DL4 (applicable for 25 species); and >70% at DL5 (applicable for 21 species) (Table 1).

**Fig 2:**
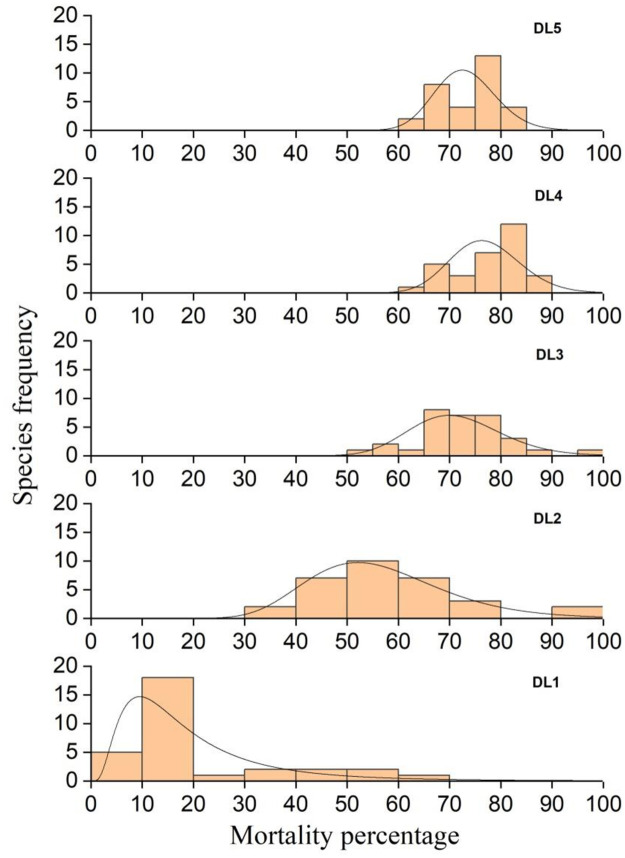
Distribution of acridid species based on mortality percentage (M%) at different density levels (DL1 to DL5). Each bar represents the number of species (Y-axis) that displayed a specific M% (X-axis). Therefore, the sum of all bars for a DL represents the total number of acridid species (n = 31). Shifting bars from lower to higher mortality scales indicates that species tend more vulnerable to death as DL increases.

Results on space-dependent mortality reflect different mortality rates of the species at different DLs. However, species distribution according to M% shows different death rates for a specific DL. For example, five species showed 0–9% mortality in DL1 (1st bar of DL1), eighteen species showed 10–19% mortality (2nd bar), only one species showed 20–29% mortality (3rd bar), two species showed 30–39% mortality (4th bar), two species showed 40–49% mortality (5th bar), two species showed 50–59% mortality (6th bar), and two species showed 60–69% mortality (7th bar) (Fig 2). This data provides generalised information about the M% of acridids for a given density concentration.

### Dose-dependent mortality

**Fig 3:**
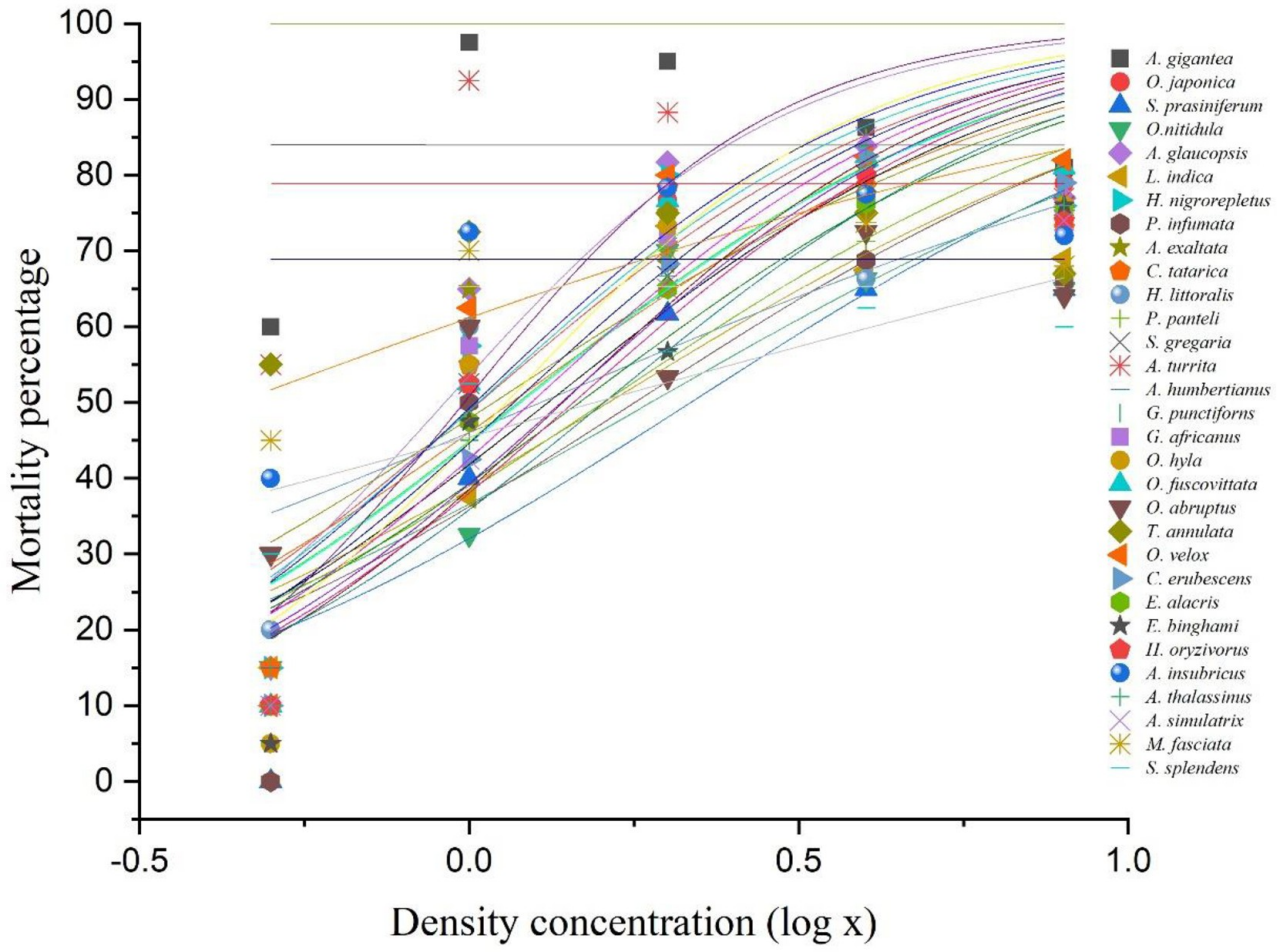
Dose-response log-logistic regression model curves for 31 acridid species. In this plot, the X-axis (independent variable) is referred to as ‘dose’ (species density in log scale = density concentration), and the Y-axis (dependent variable) is referred to as ‘response’ (mortality percentage of the species). Density concentrations (0.5, 1.0, 2.0, 4.0, and 8.0) for DL1 (10/20), DL2 (10/10), DL3 (10/5), DL4 (10/2.5) and DL5 (10/1.25) are thus plotted in log scale on the X-axis. The regression curves are developed based on species mortality at five density concentrations. The presented 31 dots for a single density concentration represent the 31 grasshopper species. The non-sigmoid mortality curves indicate that such species do not follow the rest pattern due to idiosyncratic (alike) mortalities across the DLs.

We transformed space-dependent mortality data into a dose-dependent mortality model (log-logistic dose-response model) (Fig 3) to comprehend how a DL (dose) corresponds to mortality (response) in acridids. The dose-response log-logistic regression model curves for 31 acridid species are plotted considering ‘dose’ (species density in log scale thereafter density concentration) in the X-axis (independent variable), and ‘response’ (mortality percentage of the species) in the Y-axis (dependent variable). The regression curves indicate most species followed the sigmoid functions (mathematical equations); however, only a few species do not follow the equations due to exceedingly higher mortalities at lower DLs.

**Fig 4:**
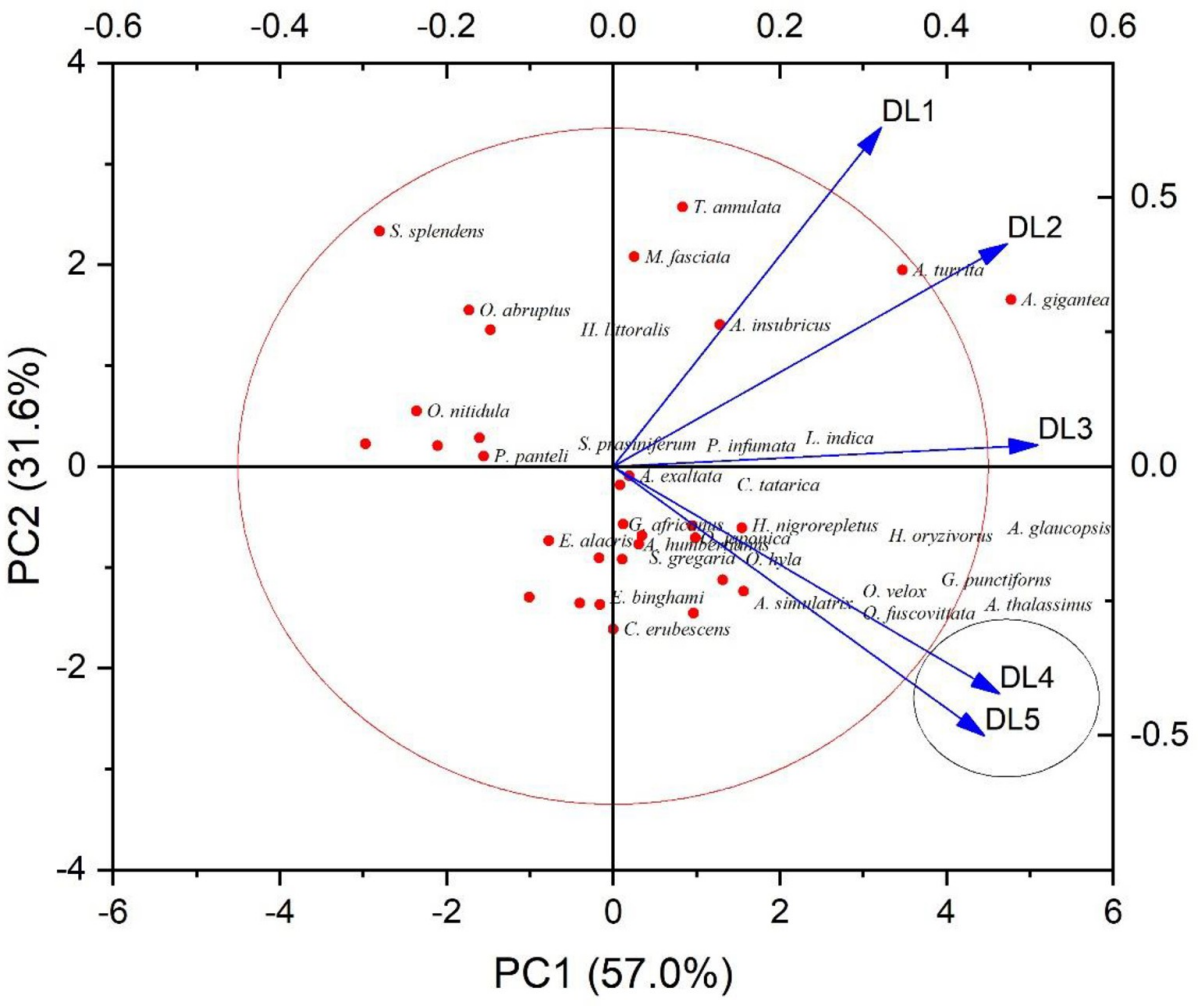
A bi-plot of principal components (PC1, 57%; PC2, 31.6%) for 31 acridid species. The PCA is classified according to the mortality percentages (M%) (response) acridids at different doses (DL1 to DL5). The cluster of DL4 and DL5 indicates a non-significant response, whereas the dispersed DLs (DL1, DL2, and DL3) indicate significant mortality changes.

**Fig 5:**
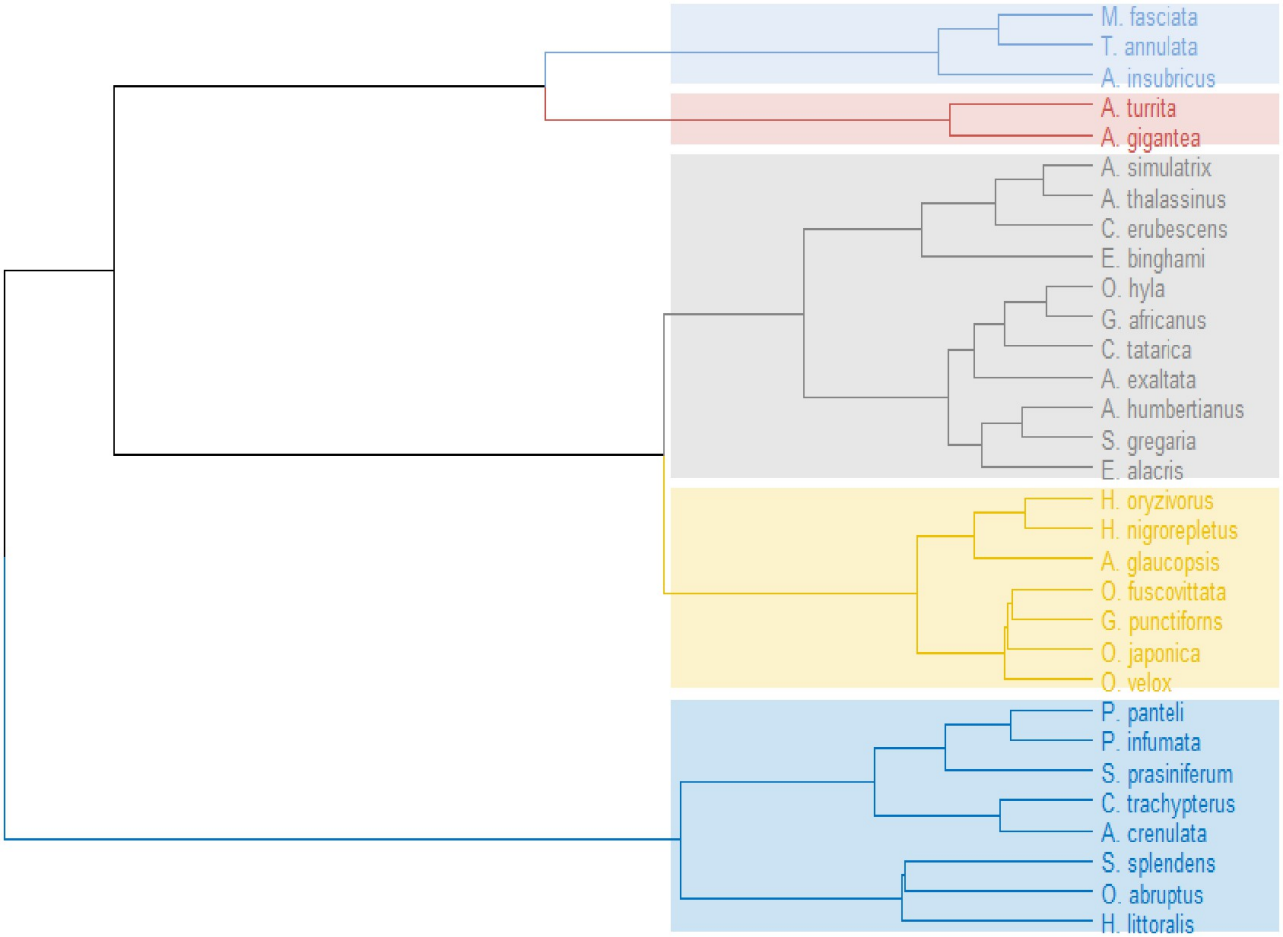
Hierarchical cluster relationship among 31 acridid species based on dose-response mortality values. The species are grouped into five clusters (color sheds) based on their differential mortality exhibitions (response). Each frond of the dendrogram corresponds to objects (species) similar to each other, merged into branches, and fused at a higher height. The higher the height of the fusion, the less similar the species are, and the less similar the species are, the higher the fusion height.

A bi-plot of principal components (PC1, 57%; PC2, 31.6%) for 31 acridid species is presented in Fig 4, where the PCA is classified according to the M% of acridids at different DLs. The cluster of DL4 and DL5 indicates a non-significant difference, where the scattered DLs (DL1, DL2, and DL3) indicate significant mortality differences among them. The principal component variables for our study are the representation of linear combinations of the original variables. Dendrogram for hierarchical cluster relationships among 31 acridid species based on dose-dependent mortality values is presented through cluster analysis. Fig 5 depicts a dendrogram for hierarchical cluster associations among 31 acridid species based on dose-dependent mortality estimates. The dendrogram objects are linked together based on similarities. Objects are connected in branches, and they are interconnected at higher levels of the dendrogram. Five clusters in the dendrogram represent the set of species with comparable mortality percentages. To determine the cluster validation, the cophenetic correlation between the cophenetic distances and the actual distances of the data shows a strong correlation (0.74). Based on space-dependent mortality, cluster-1 contains three species, cluster-2 contains two species, cluster-3 contains eleven species, cluster-4 contains seven species, and cluster-5 contains eight species, indicating that each cluster representative represents an approximately similar mortality trend in captive cultivation.

### Effective concentration

Table 2 displays the calculated effective concentrations (EC values) derived from dose-response sigmoid model functions (mathematical equation). Each species has a distinct set of values for five ECs (EC10, EC20, EC50, EC80, and EC90) which (value) indicate how mortality corresponded with densities. For example, for *O*. *japonica*, EC10 = 0.0191 indicates 10% mortality at density concentration of 0.0191. Similarly, at density concentrations of 0.03565, 0.1036, 0.30105 and 0.56186, mortality percentage of *O*. *japonica* were 20% (EC20), 50% (EC50), 80% (EC80) and 90% (EC90), respectively. In this context, density concentration is denoted as: individual number/ volume of culture space.

**Table 2:** Calculated effective concentrations (EC) for acridid species (n = 31).

Acridid species	EC10	EC20	EC50	EC80	EC90
*A*. *gigantea*	NA	NA	NA	4.94E+238	0
*O*. *japonica*	0.19103	0.35653	1.036	3.01046	5.61862
*S*. *prasiniferum*	0.22544	0.51981	2.16814	9.04334	20.85195
*O*.*nitidula*	0.14874	0.37984	1.88647	9.36913	23.92555
*A*. *glaucopsis*	0.2478	0.40191	0.91868	2.0999	3.40583
*L*. *indica*	0.15027	0.36192	1.62631	7.30797	17.60109
*H*. *nigrorepletus*	0.20814	0.37596	1.03301	2.83836	5.1268
*P*. *infumata*	0.18754	0.43057	1.78277	7.38161	16.94748
*A*. *exaltata*	0.11893	0.26933	1.08943	4.40661	9.97979
*C*. *tatarica*	0.1513	0.32066	1.15806	4.18231	8.86422
*H*. *littoralis*	0.04084	0.14581	1.28406	11.3082	40.37109
*P*. *panteli*	0.18027	0.40343	1.59887	6.33667	14.18065
*S*. *gregaria*	0.21186	0.41578	1.3165	4.16852	8.18071
*A*. *turrita*	NA	NA	NA	1.07E-87	0
*A*. *humbertianus*	0.1898	0.37333	1.18669	3.77208	7.41949
*G*. *punctiforns*	0.22233	0.3903	1.0214	2.67297	4.69235
*G*. *africanus*	0.19118	0.37628	1.19736	3.81014	7.49914
*O*. *hyla*	0.25339	0.4556	1.24217	3.3867	6.08952
*O*. *fuscovittata*	0.28939	0.47977	1.13855	2.70189	4.47933
*O*. *abruptus*	0	0	0	0	0
*T*. *annulata*	NA	NA	NA	0	0
*O*. *velox*	0.3	0.46485	0.98279	2.0778	3.21958
*C*. *erubescens*	0.29743	0.52493	1.38637	3.66146	6.46215
*E*. *alacris*	0.20948	0.42984	1.46883	5.01917	10.2992
*E*. *binghami*	0.27042	0.5214	1.60182	4.92099	9.48825
*H*. *oryzivorus*	0.23834	0.42857	1.16849	3.18591	5.7286
*A*. *insubricus*	0.00869	0.0371	0.44368	5.30567	22.65354
*A*. *thalassinus*	0.27121	0.49436	1.37965	3.85034	7.01833
*A*. *simulatrix*	0.27895	0.51116	1.43947	4.0537	7.42813
*M*. *fasciata*	NA	NA	NA	0	0
*S*. *splendens*	0.00801	0.05596	1.55228	43.05715	NA

ECs are calculated using dose-response log-logistic equation curves [EC10 = 10^ (LOGx0 –log (9)/p); EC20 = 10^ (LOGx0 + log (0.25)/p); EC50 = 10^LOGx0; EC80 = 10^ (LOGx0 + log (4)/p); EC90 = 10^ (LOGx0 + log (9)/p)]. The EC10 to EC90 values for a species describe density concentrations (dose) that cause mortality (response) ranging from 10% to 90%. NA denotes species mortality that does not follow the equation.

The tabulated EC values are represented as:

TabulatedECvalue=Individual/Spaceor,

Space=Individual/TabulatedECvalue, (1)

Individual=SpacexTabulatedECvalue. (2)

By running the tabulated EC value through Eq 1, the required space to culture for a given number of individuals can be estimated for respective species. For example, to culture 1000 individuals of *O*. *japonica* (at 10% mortality), the EC table value is 0.19103 (EC10 for *O*. *japonica*). Therefore, the required space to culture 1000 individuals of *O*. *japonica* is 1000/0.19103 or 5235 L (where a maximum of 10% adult mortality may take place during the culture period). Likewise, to culture 1000 individuals of *O*. *japonica* (at 20% mortality), the EC table value is 0.35653 (EC20 for *O*. *japonica*). And therefore, the required space to culture 1000 *O*. *japonica* is1000/0.35653 or 2805 L (where a maximum of 20% mortality may occur). Similarly, the required space to culture 1000 *O*. *japonica* can be calculated as 965 L (1000/1.036 = 965 L); 332 L (1000/3.01046 = 332 L); and 178 L (1000/5.61862 = 178 L), where to a maximum-mortality of 50%, 80%, and 90% may arise respectively. Similar calculations are applicable to all the species studied.

Likewise, by running the tabulated EC value through Eq 2, the maximum cultivable individuals can be estimated for the given available space for respective species. For example, to culture in a 1000 L space (35.31 ft^3^) a number of 191 individuals of *O*. *japonica* may be cultured where mortality may reach up to 10%. Thus, following this report, the suitable individuals for *O*. *japonica* in 1000 L space for different mortality chances are: 356 (maximum 20% mortality chance); 1036 (maximum 50% mortality chance); 3010 (maximum 80% mortality chance); and 5618 (maximum 90% mortality chance).

We were unable to calculate EC values for some species, so we left them blank (marked as not applicable, NA). This was due to the fact that such species showed exceedingly very high mortality at the DLs. For example, at DL1, species like, *A*. *gigantean* and *A*. *turrata* showed over 50% mortality (*A*. *gigantea*: 60%; *A*. *turrata*: 55%) and at DL2, they showed over 90% mortality (*A*. *gigantea*: 97.5%; *A*. *turrata*: 92.5%). Similarly, *T*. *annulate*, *M*. *fasciata*, and *A*. *insubricus* had also significantly higher mortalities than the others [DL1: *T*. *annulate* (55%), *M*. *fasciata* (45%), and *A*. *insubricus* (40%); DL2: *T*. *annulate* (72.5%), *M*. *fasciata* (70%), and *A*. *insubricus* (72%)]. Since these five grasshopper species had shown extremely high mortalities, it suggests that they require more space to survive. It was also noted that these four species did not follow the sigmoid curve, and because of getting idiosyncratic EC values, we could not generalize dose-response mortality modalities for such grasshopper species.

### BMI relation to mortality

**Fig 6:**
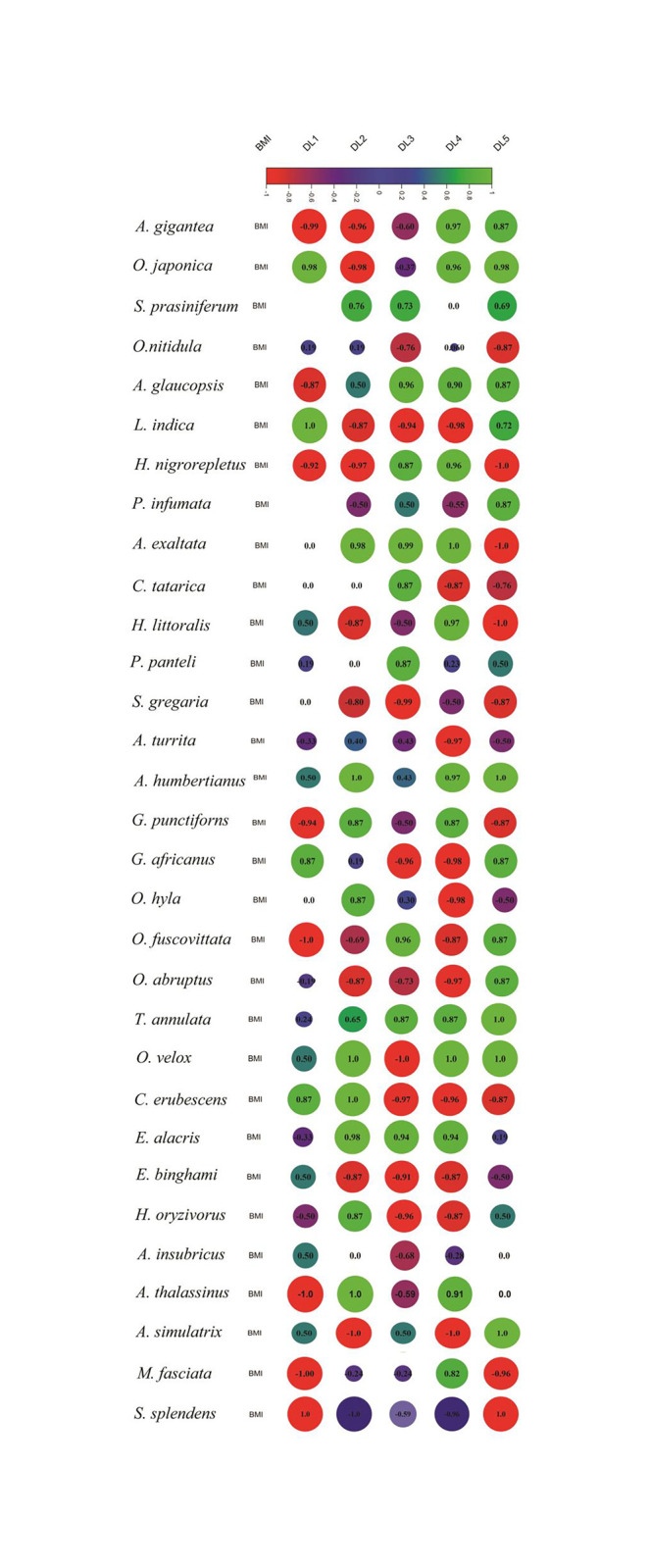
Pearson correlations between body mass index (BMI) of the species (n = 31) and mortality percentage (M%) at different density levels (DL1 to DL5). The species are aligned in ascending order of BMI (from top to bottom) and corresponding M% at each DL. Correlation coefficients (*r*) are represented by circles ranging from -1.0 (red = negatively correlated) to +1.0 (green = positively correlated). The size of the circles corresponds to the value of *r* (the bigger the circle, the higher the *r*, and vice versa). Variation of one variable related to the variation of other is referred to as *r*^2^. An *r*^2^ of 0.5 indicates 25% of variations is correlated (0.5 squared = 0.25), and thus, for *A gigantea*, *r* for -0.99 (BMI vs. DL1) indicates that BMI of *A gigantea* is 99% negatively correlated with mortality at DL1. The value ‘zero’ (*r* = 0) suggests that there is no relationship between the variables (BMI and mortality) and ‘blank’ indicates that mortality at respective DL was zero (see Table 1).

The correlation coefficients (*r*) between body-mass index (BMI) and M% of acridids for respective DL were highly variable (Fig 6). Our findings show that M% in acridids was positively correlated with BMI for only four species (*S*. *pr*. *prasiniferum*, *P*. *panteli*, *A*. *humbertianus*, and *T*. *annulata*) and negatively correlated in only one species (*S*. *gregaria*). In some, *r* changes from positive to negative scale in response to density advancement, indicating a complex relationship. For example, BMI showed positive correlation at lower DLs but became negative at higher DLs in five species (*A*. *exaltata*, *C*. *tatarica*, *O*. *hyla hyla*, *C*. *erubescens*, *E*. *binghami*). BMI, on the other hand, showed negative correlation at lower DLs but became positive at higher end for three species (*A*. *glaucopsis*, *O*. *abruptus*, *E*. *alacris alacris*). Noticeably, most of the acridids (n = 18) had irregular *r* (alternating positive to negative *r* and vice versa) across the DLs. It was hypothesised that a higher BMI ensures better survivability, but our findings contradict this assumption; instead, it states that though mortality of many acridids is BMI dependent, many of them show independent relationships.

## Discussion

Acridids have different mortality rates at different DLs, inferring that a multidimensional mortality modality exists in acridids with changing volume of space. The central tendency of our findings suggests that mortality of acridids are highly space-dependent, suggesting that cultivable space is an important parameter for acridid mass culture. The death rate, on average, rises with the advancement of the population number for a given space, but this rate may not always correspond with the individual’s size-increment. It is widely assumed that an individual’s mass is proportional to its body size [63] indicating that an individual’s survivability may be associated with the energy reserves [64] or metabolic state [65] of the organism. A species with a higher body mass is likely to have a higher energy level, and as a result, the individual with higher energy-reserves has a lower mortality. The BMI report for the 31 studied grasshopper species refers to very different morphometric patterns, and these disparities in BMIs across species may determine different mortality performances. Another theory holds that individual’s mortality is unrelated to the size of the insect [65], but rather depends on the individual’s energetics [66]. However, no clear evidence in insects supports these correlational hypotheses.

We observed variable mortalities in acridids for a specific DL despite the fact that we did not perform any biochemical (fat or fat-free body reserves) or bioenergetics tests for our species to add additional knowledge about space-dependent mortality. In order to establish a general trend of mortality performance among acridids for specific DL-conditional space, we compiled data for all species (n = 31) mortality-values for a DL, and calculated the mean mortality percentage of all species for that DL. The mean (±SD) mortalities of acridids for each DL were 18.54±16.13 (DL1); 56.69±14.14 (DL2); 71.55±8.91 (DL3); 77.09±6.59 (DL4) and 73.16±5.79 (DL5). Though this information does not explicitly confirm the best species to culture under a specific DL, it does provide an imprecise update on the rearing unit capacity of acridids for mass-culture. Our results clearly show that mortality of acridids increases significantly from low to highly packed DL up to a certain threshold, but then mortality does not increase significantly. The mortality percentage of acridids increases significantly from DL1 to DL2 and then from DL2 to DL3, but there is no significant change in mortality from DL3 to DL4 or from DL4 to DL5. As a result, with the exception of a few, most of the studied species followed a sigmoidal dose-response model curve. Principal component analysis (PCA) (Fig 4) also agrees that DL4 and DL5 cluster together with non-significant differences, whereas the remaining three DLs (DL1, DL2, and DL3) have significant differences in mortality values. A dendrogram plot generated by species clustering (Fig 5) reveals at least 5 clusters of acridid species with different mortality percentages.

Five species, such as *A*. *gigantea* (60%, 97.5%, 95%, 86.25%, and 81%); *T*. *annulate* (55%, 72.5%, 75%, 75%, and 67%); *A*. *turrita* (55%, 92.5%, 83.33%, 81.25%, and 77%); *M*. *fasciata* (45%, 70%, 70%, 73.75%, and 68%), and *A*. *insubricus* (40%, 72.5%, 78.33%, 77.5% and 72%) showed higher mortality percentages at DL1, DL2, DL3, DL4, and DL5 respectively (stated in parenthesis). Since these species have a greater mortality rate (around 50% at DL1), it suggests that they require more rearing space in order to have a lower mortality rate. As we chose a uniform setup for all species, we calculated ECs correspondingly. It was noted that these four species did not follow the sigmoid curve due to very high mortality rates, even reared at larger spaces. Our result indicates that grasshoppers such as *A*. *gigantea*, *T*. *annulate*, *A*. *turrita*, *M*. *fasciata*, and *A*. *insubricus* are not suitable for mass culture due to their high mortality rate.

The analysis for independent contrasts of required space and BMI of studied species has not been purposefully depicted in our text; instead, we focus on the mortality assay of adult acridids under altered space situations. We did not investigate the insects’ growth rate or cannibalism, which could affect individual survivability. Our findings describe the mortality performance of grasshopper species at various density levels, allowing us to develop a general dose-response model for the species. As a result, the current study provides an overall picture of the space requirements for mass culture of acridids in captivity. A utility chart depicting independent ‘concentration value’ (EC) of required space for thirty-one studied species has been prepared as a ready reference for grasshopper mass-culture. The projected chart includes the conversion factor needed to estimate space for a given number of grasshoppers. In other words, the projected chart will assist in estimating the number of individuals to be cultured for a given space. Despite the fact that our chart value addresses species-specific information, it largely provides an inclusive picture of other species. As space is directly related to the survivability (and mortality) of cultivable organisms, it has a direct influence on crop yield (insect biomass), and thus our findings may be useful for the smooth propagation of insect mass culture, particularly for the acridids. However, additional parameters such as abiotic factors, particularly rearing temperature and relative humidity; life-history traits of cultivable species such as breeding frequency, fecundity, and fertility; and other beneficial production traits such as growth rate and lifespan of the specific species should be standardized concurrently with density in order to define the optimal rearing conditions for maximal yield.

## Supporting information

S1 Table This supplement is for ’Fig-3’ dose-response log-logistic regression model. It contain the mortality value with respect to log x value.
(XLS)

S2 Table This supplement is for ’Fig-4’ of principal components analysis. It contains loading plot data and eigenvalues.
(XLS)
